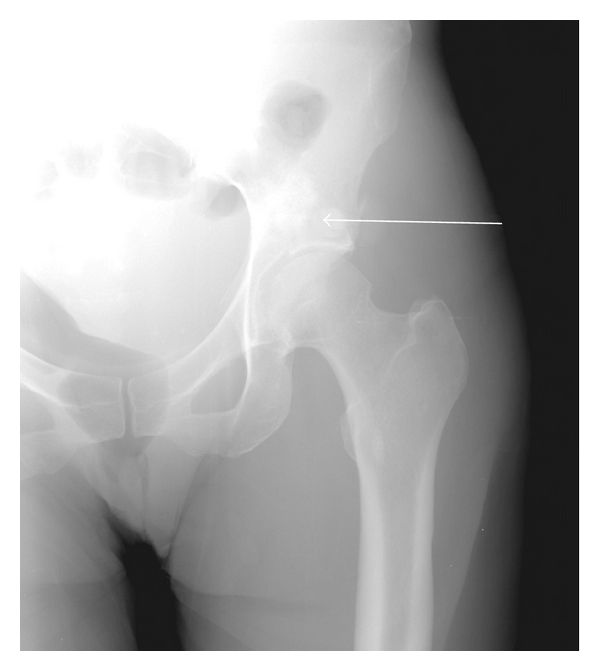# POEMS Syndrome Diagnosed 10 Years after Disabling Peripheral Neuropathy

**DOI:** 10.1155/2011/126209

**Published:** 2011-10-15

**Authors:** Viet H. Nguyen

**Affiliations:** Department of Internal Medicine, Cleveland Clinic Florida, 2950 Cleveland Clinic Boulevard, Weston, FL 33331, USA

## Abstract

Peripheral neuropathy is characterized as a generalized, relatively homogeneous process affecting many peripheral nerves and predominantly affecting distal nerves. The epidemiology of peripheral neuropathy is limited since the disease presents with varying etiology, pathology, and severity. Toxic, inflammatory, hereditary, and infectious factors can cause damage to the peripheral nerves resulting in peripheral neuropathy. Peripheral neuropathy is most commonly caused by diabetes, alcohol, HIV infection, and malignancy. We report a case of a 42-year-old female with 10-year history of progressively worsening peripheral neuropathy, hypothyroidism, and skin changes who presents with dyspnea secondary to recurrent pleural and pericardial effusions. Prior to her arrival, her peripheral neuropathy was believed to be secondary to chronic demyelinating inflammatory polyneuropathy (CDIP) given elevated protein in the cerebral spinal fluid (CSF) which was treated with intravenous immunoglobulin (IVIG) and corticosteroids. Unfortunately, her peripheral neuropathy did not have any improvement. Incidentally, patient was found to have splenomegaly and papilledema on physical exam. Serum protein electrophoresis showed a monoclonal pattern of IgA lambda. Patient met the diagnostic criteria for POEMS (polyneuropathy, organomegaly, endocrinopathy, M-protein, and skin changes) syndrome. An underlying diagnosis of POEMS syndrome should be considered in patients with chronic debilitating neuropathy and an elevated protein in the CSF.

## 1. Case Presentation

**Figure 1:**
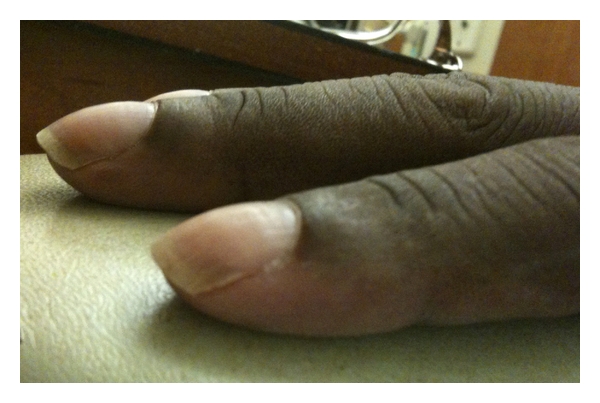


**Figure 2:**
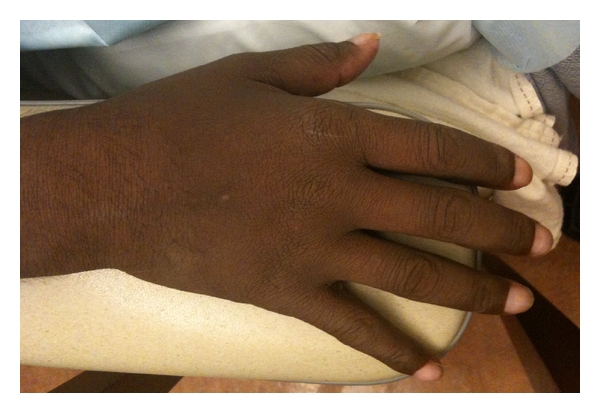


**Figure 3:**
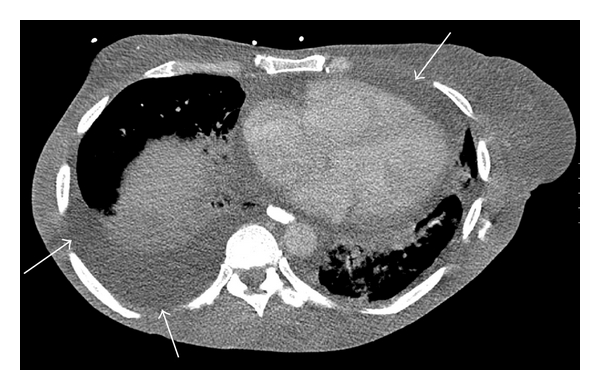


**Figure 4:**
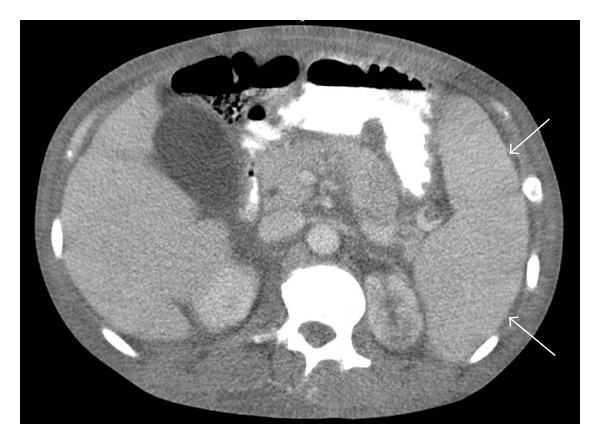


A 42-year-old South Asian female with a history of 10 years of disabling peripheral neuropathy treated with corticosteroids and intravenous immunoglobulin (IVIG) without improvement. About 1 year ago, she developed hypertrichosis, skin hyperpigmentation, and nail clubbing and whitening (nail clubbing, Figure 1; hypertrichosis and nail whitening, Figure 2). She also developed hypothyroidism 1 year ago. Evaluation of a headache 1 year ago revealed that the patient had bilateral papilledema. Complete blood count showed a white blood count of 7.8, hemoglobin of 10.4 g/dL, hematocrit of 31.9%, and platelets of 306. Cerebral spinal fluid analysis from a lumbar puncture showed a total protein of 63 g/dL. Patient was presumed to have CDIP. Serum protein electrophoresis showed a monoclonal pattern of IgA lambda. She also began to have to functional decline and significant weight loss for the past 2 years. Computed tomography (CT) of the chest showed pericardial effusion and bilateral pleural effusions (Figure 3, arrows). For the past 3-4 months, she developed dyspnea secondary to recurrent pleural effusions and pericardial effusions requiring multiple thoracenteses and pericardiocentesis, respectively. A CT Scan of the abdomen and pelvis confirmed splenomegaly and ascites (Figure 4, arrow). A whole body skeletal survey revealed osteosclerotic lesions of the left iliac bone and proximal left femur (Figure 5, arrow). By piecing together distinct signs and symptoms, the most likely diagnosis is POEMS syndrome. There is no single laboratory test that can be performed to diagnose POEMS syndrome. Multiple signs and symptoms are associated together to establish the diagnosis of POEMS syndrome. Two major criteria: polyneuropathy and monoclonal plasmaproliferative disorder and at least 1 minor criterion: osteosclerotic myeloma, Castleman's disease, organomegaly, endocrinopathy (excluding diabetes mellitus or hypothyroidism), edema, typical skin changes, and papilledema are required for diagnosis of POEMS syndrome defined by a retrospective review at the Mayo Clinic [1]. The patient exhibits both major criteria and many of the minor criteria (sclerotic bone lesions, organomegaly, edema or effusions, skin changes, papilledema, and endocrinopathy).

## 2. Pathogenesis

The exact cause of POEMS syndrome is poorly understood, although proinflammatory cytokines have been implicated as a cause of the disease [2–4]. Patients with POEMS syndrome had higher serum levels of IL-1 beta, TNF-alpha, and IL-6 and lower serum levels of TGF beta 1 than did patients with multiple myeloma [5]. Vascular endothelial growth factor (VEGF) is a good pathogenic marker in POEMS [6, 7]; it induces a rapid and reversible increase in vascular permeability and neovascularization. The overproduction of VEGF is important in the pathogenesis of POEMS syndrome. Our patient had an Interleukin-6 level of 26.3 pg/mL (0–14 pg/mL) and a VEGF level of 1960 pg/mL (31–86 pg/mL). After chemotherapy and autologous stem cell transplantation, the symptoms of the syndrome improved along with the decrease in serum VEGF levels [8, 9].

The median age of patients diagnosed with POEMS syndrome is 51 years of age [1]. Peripheral neuropathy is a significant feature of the syndrome and a required criterion for diagnosis. The peripheral neuropathy is ascending, with either an insidious or rapidly progressing onset. Patients often initially have numbness and dysesthesias which is then followed by a progressive ascending weakness that overshadows the sensory impairment. Increased cerebral spinal fluid protein was seen in all the patients from a case series. Sixty-seven to eighty-four percent of patients will have an endocrine abnormality at presentation with hypogonadism being the most common and fifty percent of the patients had organomegaly (hepatomegaly, splenomegaly, and lymphadenopathy). Two-thirds of the patients had skin changes (hyperpigmentation, hypertrichosis, acrocyanosis, plethora, and hemangioma/telangiectasia). 

A retrospective case series showed overall median survival was 165 months. There has not been a documented case of POEMS syndrome in a South Asian American prior to this case report. Previously, there were case series in Chinese patients, Japanese patients, and non-Asians [10]. Prognostic markers for survival included fingernail clubbing and extravascular fluid overload (edema, effusion, or ascites). Patients with clubbing or volume overload had a median survival of 31 and 79 months, respectively [1].

More than half the patients respond to radiation therapy with nonneurologic manifestations improving prior to neurologic manifestations. Patients with a solitary lesion or a dominant lesion are treated with radiation therapy as first-line therapy. The hypothesis implicating vascular endothelial growth factor [6–8] eventually may be validated, providing a uniform target for therapy. Systemic and skin symptoms tend to respond sooner than do symptoms of peripheral neuropathy, with the former beginning to respond within 1 month, and the neuropathy responds within 3 to 6 months. Many treatment strategies other than radiation therapy have been used, including plasmapheresis, intravenous immunoglobulin, interferon alfa, corticosteroids, alkylators, azathioprine, autologous stem cell transplantation (ASCT), tamoxifen, and transretinoic acid. The current mainstay treatment for POEMS is radiation therapy and alkylator-based therapy [11]. There have been promising results with the use of high-dose chemotherapy with melphalan and autologous blood stem cell transplantation.

## 3. Conclusion

There is vast spectrum of causes of peripheral neuropathy, which commonly include diabetes, alcohol abuse, and HIV infection. Our patient suffered from 10 years of chronic peripheral neuropathy, and she had an extensive workup by multiple physicians. Ultimately, she was diagnosed with POEMS syndrome, a rare paraneoplastic syndrome from a plasma cell dyscrasia, which most commonly presents with peripheral neuropathy. Although uncommon, POEMS syndrome should be considered among patients with chronic peripheral neuropathy. 

## Figures and Tables

**Figure 5:**